# Phagocyte NADPH Oxidase NOX2-Derived Reactive Oxygen Species in Antimicrobial Defense: Mechanisms, Regulation, and Therapeutic Potential—A Narrative Review

**DOI:** 10.3390/antiox15010055

**Published:** 2025-12-31

**Authors:** George Țocu, Bogdan Ioan Ștefănescu, Loredana Stavăr Matei, Lavinia Țocu

**Affiliations:** 1 Department of Pharmaceutical Sciences, Faculty of Medicine and Pharmacy, “Dunarea de Jos” University, 800008 Galati, Romania; george.tocu@ugal.ro; 2 Department of Clinical Surgery, Faculty of Medicine and Pharmacy, “Dunarea de Jos” University, 800008 Galati, Romania; 3 Department of Clinical Medicine, Faculty of Medicine and Pharmacy, “Dunarea de Jos” University, 800008 Galati, Romania; 4 Department of Clinical Cardiology, “Sf. Apostol Andrei” County Emergency Clinical Hospital, 800578 Galati, Romania; lavinia.tocu@ugal.ro

**Keywords:** reactive oxygen species, NADPH oxidase, innate immunity, infection, oxidative stress, redox signaling, antimicrobial therapy

## Abstract

ROS derived from NADPH oxidase, particularly NOX2, are central to antimicrobial defense, coupling direct pathogen killing with redox signaling that shapes inflammation. This narrative review integrates recent advances on NOX2 structure, assembly, and spatiotemporal control in phagocytes, and outlines how ROS interact with NF-κB, MAPK, and Nrf2 networks to coordinate microbicidal activity and immune modulation. We summarize evidence that both ROS deficiency, as in chronic granulomatous disease, and uncontrolled excess, as in sepsis and severe COVID-19, drive clinically significant pathology, emphasizing the need for precise redox balance. Emerging therapeutic strategies include selective NOX2 inhibitors that limit pathological oxidative bursts, redox-modulating peptides that disrupt upstream activation cues, and Nrf2 activators that enhance endogenous antioxidant capacity, with attention to dosing challenges that preserve host defense while mitigating tissue injury. Key gaps remain in biomarker standardization, real-time in vivo ROS monitoring, and translation from animal models to patients, motivating personalized, combination approaches to redox medicine in infectious diseases.

## 1. Introduction

Reactive oxygen species (ROS) generated by the NADPH oxidase complex constitute a central pillar of innate antimicrobial defense, ensuring both the direct destruction of invading pathogens and the orchestration of protective immune signaling [1]. In phagocytic cells (such as neutrophils and macrophages), the NOX2 isoform of NADPH oxidase catalyzes the transfer of electrons from NADPH to oxygen, forming superoxide (O_2_•^−^). Superoxide is rapidly dismutated to hydrogen peroxide (H_2_O_2_), which is subsequently converted by myeloperoxidase into hypochlorous acid (HOCl), a potent oxidant with direct microbicidal effects [1]. This phagocytic oxidative “burst” efficiently destroys ingested bacteria and fungi; its relevance is underscored by the fact that inherited deficiencies of the NOX2 complex cause chronic granulomatous disease (CGD), characterized by extreme susceptibility to recurrent infections and granulomatous inflammation [2].

Conversely, excessive or uncontrolled ROS production can cause cellular and tissue injury, contributing to inflammatory and autoimmune pathologies and underscoring the need for tight regulation of NADPH oxidase activity [3]. Activation of the NOX2 complex is finely controlled by signaling-dependent mechanisms; for example, phosphorylation of the cytosolic p47phox subunit and recruitment of Rac GTPases are essential for subunit translocation to the membrane and assembly of the active complex only in the presence of infectious stimuli (chemokines, PAMPs, etc.) [4]. Thus, the immune system leverages ROS in two complementary ways: as cytotoxic weapons against microbes and as signaling mediators that tune inflammatory responses and modulate immune cell function [5]. A complete absence of ROS (as in CGD) disrupts these processes and leads to severe immunodeficiency, whereas excessive ROS levels can trigger pathological oxidative-stress pathways, highlighting the importance of maintaining a homeostatic redox balance during the response to infection [6].

Given the central role of NOX2 derived ROS in antimicrobial defense, several lines of research have emphasized how oxidative mechanisms coordinate pathogen killing and immune signaling. Recent studies highlight that NOX2 dependent ROS not only drive microbicidal activity within phagolysosomes but also regulate cytokine production, phagocyte recruitment, and the resolution of inflammation [7,8]. Defects in this pathway, as observed in genetic or acquired NOX2 impairment, markedly reduce microbial clearance and predispose to severe infections, confirming the essential contribution of NOX2 to host defense [9,10,11]. Conversely, excessive or dysregulated ROS generation amplifies tissue injury and inflammatory damage, a phenomenon documented in diverse infectious settings, including acute bacterial infections and hyperinflammatory responses [12,13,14]. These observations underscore the need to understand the molecular architecture and regulation of NOX2 in greater detail, as balanced ROS output is indispensable for effective antimicrobial immunity. Building on these concepts, the present review focuses on the mechanisms by which the NOX2 complex generates and controls ROS, how these oxidants modulate immune pathways, and how dysregulation contributes to infectious pathology.

## 2. Materials and Methods

### 2.1. Article Type and Methodological Guidance

This narrative review focused on the role of ROS derived from NADPH oxidase in antimicrobial defense. We followed good practices for transparency, including explicit specification of the search strategy, selection criteria, and critical appraisal of the evidence.

### 2.2. Data Sources and Search Strategy

Searches were performed in PubMed/MEDLINE, Scopus, and the Web of Science Core Collection, gray literature was consulted selectively via Google Scholar to identify frequently cited articles that did not appear in the initial queries. The time window for study selection was January 2015 through October 2025, last update 14 October 2025, with an emphasis on studies published between 2020 and 2025, and earlier works were included only when essential for conceptual or methodological grounding. We used combinations of free-text terms and subject headings where available, for example, in PubMed: (“NADPH oxidase” OR NOX2 OR CYBB OR “gp91phox” OR “phagocyte oxidase” OR NOX1 OR NOX3 OR NOX4 OR NOX5 OR DUOX*) AND (“reactive oxygen species” OR ROS OR superoxide OR “oxidative burst”) AND (infection* OR pathogen* OR antimicrobial OR “host defense” OR phagocyt* OR “neutrophil extracellular traps” OR NETs). For the therapeutic component, we added terms such as: (inhibitor* OR activator* OR modulator* OR “pharmacologic*” OR “cold atmospheric plasma” OR CAP OR PAM OR “photodynamic therap*” OR PDT OR nanoparticle* OR nanomaterial*). References in key articles were also tracked, snowballing, to identify additional relevant studies.

### 2.3. Inclusion and Exclusion Criteria

Inclusion: (I) peer reviewed original studies, reviews, and meta-analyses that address the mechanisms, regulation, or therapeutic implications of NADPH oxidase derived ROS in infectious contexts; (II) human, animal, or cellular models with translational relevance; (III) articles in English.

Exclusion: (I) works focused exclusively on oncology or wound healing without an infectious component; (II) non mammalian systems without clear relevance to human antimicrobial defense; (III) editorials or commentaries without data; (IV) non-peer-reviewed preprints; (V) retracted or duplicate articles. Exceptionally, CGD case reports, chronic granulomatous disease, were included if they provided mechanistic clarification regarding NOX2 deficiency.

### 2.4. Study Selection and Data Extraction

Selection was performed in two steps, title and abstract screening, then full text assessment for eligibility. From each relevant study we extracted, the NOX isoform involved, the model and pathogen type, the ROS quantification method, for example, luminol, HyPer probe, cytochrome c, antimicrobial endpoints, for example, CFU reduction, bacterial or fungal killing, and interactions with Myeloperoxidase (MPO) or Neutrophil extracellular traps (NETs), and for clinical studies, the patient phenotype, for example, CGD, the infection site, the redox intervention, and the outcomes.

### 2.5. Quality Assessment and Narrative Synthesis

Because this is a narrative review, we did not apply a single formal risk of bias scale, we prioritized studies with rigorous design, replication, appropriate controls, and adequate sample size, the convergence of results across models, in vitro, in vivo, and clinical, and mechanistic plausibility. The synthesis was thematic, organized by mechanisms, redox regulation, and therapeutic potential, with emphasis on gaps and future directions.

## 3. NADPH Oxidase Family and Structure

Although the NADPH oxidase family comprises several isoforms, this review focuses on the phagocyte enzyme NOX2, which is the prototypical antimicrobial oxidase in innate immunity.

The NOX family (NOX1–5 and DUOX1/2) consists of membrane-bound flavoproteins that share a conserved catalytic core but differ in regulatory partners and activation mechanisms [15,16]. NOX1, NOX3, and NOX2 form heterodimers with p22phox and require cytosolic cofactors for activation, whereas NOX5 and DUOX1/2 possess calcium-binding EF-hand domains, with DUOX enzymes also containing a peroxidase-like region [16]. Isoform distribution is tissue-specific, for example, NOX1 in epithelial and vascular cells, NOX3 in the inner ear, NOX4 in kidney and endothelium, NOX5 in lymphoid tissues, and DUOX1/2 in mucosal epithelia where they generate H_2_O_2_ for barrier defense [17]. The following sections focus exclusively on NOX2 structure, regulation, and antimicrobial function.

NOX2 and the Phagocyte Oxidase Complex: NOX2, also known as the classic phagocyte NADPH oxidase, is the prototype of the NOX family and plays a central role in innate antimicrobial defense [15]. It is chiefly expressed in professional phagocytes, neutrophils, monocytes or macrophages, and dendritic cells, with neutrophils showing the highest NOX2 activity, the “respiratory burst,” upon activation [16]. Lower levels are present in other cells (e.g., endothelial and muscle cells), but in immune cells NOX2-derived ROS represent a primary microbicidal mechanism [18]. Indeed, NOX2 is indispensable for host defense: loss-of-function in any component of the NOX2 system (due to genetic defects) causes chronic granulomatous disease (CGD), characterized by recurrent, severe infections due to an impaired oxidative burst [19]. For many years, the only known role of NOX2 was pathogen killing, and it remains the paradigmatic example of ROS-based immunity [15].

NOX2 operates as a multi-component enzyme complex, with membrane-bound and cytosolic subunits that assemble dynamically [19]. The membrane portion, known as flavocytochrome b558, is a stable heterodimer composed of the NOX2 catalytic subunit (gp91phox) and the smaller p22phox subunit in 1:1 stoichiometry [15]. NOX2/gp91phox contains the electron-transporting machinery: its N-terminal transmembrane region (six helices) embeds two b-type hemes that conduct electrons across the membrane, while its C-terminal cytosolic region (ferrodoxin-NADP^+^ reductase homology) binds flavin adenine dinucleotide (FAD) and NADPH [20]. The p22phox subunit is essential for NOX2 stability and function. Each stabilizes the other during biosynthesis, and the absence of either prevents the complex from reaching the membrane [15]. Functionally, p22phox acts as a scaffold; it not only tethers NOX2 in the membrane but also provides an docking site (a proline-rich cytosolic tail) for organizer proteins like p47phox upon activation [21]. In resting phagocytes, the NOX2/p22 heterodimer remains dormant in the plasma or granular membrane, awaiting assembly with its cytosolic partners [15].

The regulatory subunits of the phagocyte oxidase, p47phox, p67phox, and p40phox, reside in the cytosol as an inactive complex together with the small GTPase Rac when the cell is not stimulated [22]. p47phox (the “organizer”) and p40phox both contain PX domains that bind membrane phospholipids and Src Homology 3 domain (SH3 domains), but in resting cells they are autoinhibited by intramolecular interactions [19]. p67phox (the “activator”) has domains for binding Rac and for directly stimulating NOX2’s catalytic core [23]. Upon pathogen encounter or inflammatory stimulus, a kinase signaling cascade (e.g., Protein Kinase C—PKC, Mitogen-Activated Protein Kinases—MAPKs) phosphorylates multiple serines on p47phox (and p40phox), releasing its autoinhibition [24]. This conformational change exposes the SH3 domains of p47phox, enabling it to bind the proline-rich region (PRR) of p22phox on the membrane [19]. Concurrently, the GTPase Rac (Rac2 in neutrophils) is activated by GTP loading and translocates to the membrane, where it interacts with p67phox (via p67’s tetratricopeptide repeat domain) and also contacts the NOX2 catalytic subunit [25]. The cytosolic complex (p47–p67–p40 and Rac-GTP) thus translocates and assembles with NOX2/p22phox at the membrane, forming the competent oxidase [25]. This assembly is tightly regulated and typically occurs at the phagosome membrane enclosing an ingested microbe, although certain stimuli (e.g., chemokines, TLR agonists) can induce assembly at the plasma membrane to release ROS extracellularly [26]. Notably, the multi-step assembly requirement ensures that NOX2 remains inactive until appropriately triggered, preventing unwarranted oxidative damage to host tissues [26].

Activation of the phagocyte NADPH oxidase (NOX2) complex begins when pathogen recognition induces phosphorylation of cytosolic subunits, most notably p47phox, which releases autoinhibition and allows the p47–p67–p40 complex together with Rac2 to translocate to the membrane. There, these components assemble with the flavocytochrome b558 (NOX2/p22phox heterodimer) to form the active enzyme [15,27]. Once assembled, NOX2 transfers electrons from cytosolic NADPH to molecular oxygen through FAD and the two heme groups, generating superoxide within the phagosome [28,29,30]. Superoxide rapidly dismutates into H_2_O_2_, which can subsequently be used by myeloperoxidase to generate additional microbicidal oxidants [28,29]. These ROS collectively drive the oxidative burst that enables degradation of engulfed pathogens and also support redox-dependent modulation of inflammatory signaling and neutrophil recruitment [31]. The essential nature of this system is illustrated by chronic granulomatous disease, where inherited defects of NOX2 subunits impair ROS generation and lead to recurrent infections accompanied by dysregulated inflammation [32].

## 4. Mechanisms of ROS Production in Host Defense

The phagocyte NADPH oxidase NOX2 is the main generator of ROS in neutrophils and macrophages, producing the rapid oxidative burst required for microbial killing [33]. Defective activation of this system, due to impaired NOX2 assembly or signaling, compromises pathogen elimination and contributes to inflammatory dysregulation [16]. The enzyme complex consists of the membrane-bound gp91phox/p22phox heterodimer and the cytosolic subunits p47phox, p67phox, p40phox, and Rac, which assemble at the phagosomal membrane upon stimulation [34]. Once formed, NOX2 transfers electrons from cytosolic NADPH to molecular oxygen within the phagosome, generating superoxide [35]. Confining this reaction to the phagocytic compartment ensures that oxidants are concentrated on internalized microbes while limiting collateral injury to surrounding tissues [36]. Superoxide then dismutates to H_2_O_2_, which serves both as a diffusible oxidant and as substrate for granule enzymes such as myeloperoxidase. In neutrophils, MPO uses H_2_O_2_ to form HOCl, a potent antimicrobial oxidant that greatly amplifies the microbicidal capacity of the oxidative burst [37].

At the phagolysosomal level, ROS directly attack the engulfed microorganisms. Superoxide, and especially the more diffusible derived species, H_2_O_2_, HOCl, the hydroxyl radical (•OH), can traverse microbial membranes and oxidize essential components, nucleic acids, proteins, and lipids, causing lethal damage to the pathogen [35]. In macrophages, superoxide produced by NOX2 can react with nitric oxide (NO) generated by inducible nitric oxide synthase (iNOS), forming peroxynitrite (ONOO^−^), another cytotoxic oxidant that contributes to intraphagocytic bactericidal activity [37].

Beyond their microbicidal effects, ROS function as signaling mediators in the innate immune response. For instance, in neutrophils, ROS generated by the NADPH oxidase complex can activate granular proteases and induce the formation of neutrophil extracellular traps (NETs) [35]. At the molecular level, H_2_O_2_ that diffuses into the cytosol can oxidize critical cysteine residues in signaling proteins, thereby modulating transduction pathways (e.g., NF-κB, MAPK) and the expression of immune genes according to the cell’s redox state. These immunomodulatory roles of ROS require strict regulation, insufficient ROS production predisposes to severe infections, whereas uncontrolled excess provokes exaggerated inflammation and collateral tissue injury [38]. Physiologically, phagocytes control the temporal and spatial release of ROS, for example, by confining oxidative “bursts” to the interior of phagosomes, to maximize the antimicrobial effect and minimize oxidative stress on the host [38].

Other cellular sources of ROS in infection also include:

- Mitochondria. The mitochondrial electron transport chain generates ROS, primarily superoxide, as a byproduct of oxidative phosphorylation, and these represent an important source of ROS in eukaryotic cells [39]. Recent studies show that the production of mitochondrial ROS (mtROS) can be activated by immune signals, for example, through recognition of PAMPs (pathogen-associated molecular patterns) or exposure to proinflammatory cytokines, which suggests a direct role for mtROS in antimicrobial defense [33]. Such mitochondrial ROS can contribute to inflammasome activation and to the amplification of antipathogen immune signaling.
- Xanthine oxidase. The enzyme xanthine oxidase (XO), involved in purine catabolism, generates superoxide and H_2_O_2_ as byproducts of the oxidation of hypoxanthine and xanthine to uric acid. Therefore, XO constitutes an additional source of ROS in inflammatory contexts, and experimental models have shown that ROS derived from XO can activate the inflammasome and contribute to defense against certain infections, for example, in parasitic infections [40].
- Microbial enzymes and microbially derived ROS. Some microorganisms produce ROS themselves, influencing the ecology and pathogenesis of infection. For example, *Streptococcus pneumoniae*, which does not express catalase, generates millimolar amounts of H_2_O_2_ through the activity of a flavin oxidase, the pyruvate oxidase SpxB [41]. This endogenous H_2_O_2_ helps the pneumococcus inhibit other bacteria in the nasopharyngeal flora and exerts cytotoxic effects on host cells, for example, on respiratory epithelial cells and neutrophils [41]. Conversely, many pathogenic bacteria possess antioxidant enzymes, catalases, peroxidases, and superoxide dismutases, which protect them from host generated ROS. For instance, *Staphylococcus aureus* shows notable resistance to oxidative killing because it expresses both catalase and superoxide dismutase, which neutralize H_2_O_2_ and superoxide produced by neutrophils [42].

Overall, the NOX2/MPO system and the ROS flux generated in phagocytes constitute a central effector mechanism of antimicrobial immunity, capable of directly destroying pathogens through oxidation and, at the same time, modulating the inflammatory cascade through redox signaling [42]. Fine regulation of these processes, through phagosomal compartmentalization and through balance with the host’s antioxidant systems, is essential for effective defense against infections while minimizing damage to host tissues.

## 5. NOX2-Dependent Redox Regulation of Immune Signaling Pathways

Although numerous studies, including comprehensive reviews, have addressed redox signaling in immune cells, key mechanistic insights into NOX2 activation and redox-dependent regulation of inflammatory pathways are derived from seminal biochemical and genetic studies, which are highlighted here alongside selected review articles to provide integrative context.

### 5.1. Activation and Regulation of NOX2 in Phagocytes

**Figure 1:**
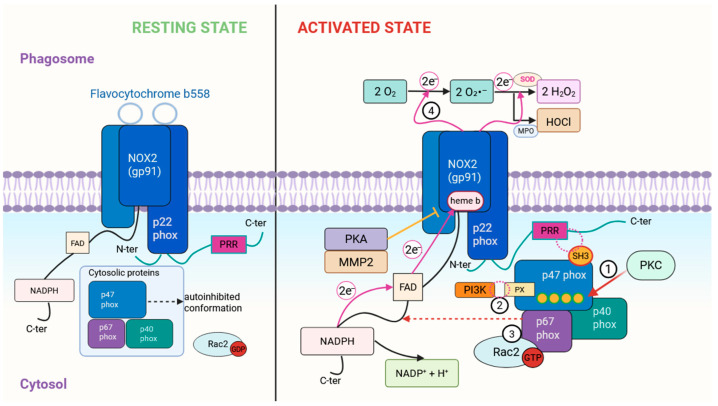
Schematic representation of NOX2 activation in phagocytes. In the resting state, the NOX2–p22phox flavocytochrome is embedded in the phagosomal membrane, while cytosolic subunits remain inactive. Upon stimulation, PKC phosphorylates p47phox (Step 1), relieving autoinhibition and exposing its SH3 domains. These SH3 domains bind the PRR of p22phox, initiating membrane recruitment of the p47–p67–p40 complex. Phosphoinositide 3-Kinase (PI3K)-derived phosphoinositides further stabilize this interaction through engagement of the PX domain of p47phox (Step 2). Activated Rac2-GTP then translocates to the membrane and recruits p67phox, promoting assembly of the functional oxidase together with p40phox (Step 3). Through its interaction with p67phox and the cytosolic catalytic domain of gp91phox, Rac2 facilitates initiation of the electron-transfer reaction. Once assembled, NOX2 transfers electrons from cytosolic NADPH, via FAD and the transmembrane heme groups, to molecular oxygen, generating superoxide within the phagosomal lumen (Step 4). Negative regulators such as Protein Kinase A (PKA) and Matrix Metalloproteinase-2 (MMP2) attenuate NOX2 activity.

This section summarizes the molecular sequence that activates NOX2 in phagocytes, from the resting architecture to assembly of the active complex and negative regulation, as illustrated in Figure 1.

In phagocytes, the transition from the resting to the activated state involves rapid post-translational regulation of the cytosolic NOX2 subunits. Upon stimulation, PKC-dependent phosphorylation of p47phox relieves its autoinhibited conformation and enables membrane targeting through binding to the PRR of p22phox [2,43]. Concurrently, phosphoinositide generation by PI3K stabilizes this membrane recruitment by engaging the PX domains of cytosolic proteins [44]. Activation of Rac2, which translocates to the phagosomal membrane, further promotes functional assembly by interacting with p67phox and facilitating the conformational changes required for electron transfer [25]. Through these coordinated steps, phagocytes achieve rapid on-demand oxidase activation without redundant expression changes, ensuring that ROS generation occurs only under appropriate inflammatory or microbial cues. GTP–Rac2 interacts with p67phox (helping recruit p67phox/p47phox to gp91phox) and also binds directly to gp91phox, promoting conformational changes that trigger electron transfer [45]. Through this well-orchestrated mechanism, NOX2 is activated to transfer electrons from cytosolic NADPH to molecular oxygen, generating superoxide within the phagosome or at the plasma membrane. This respiratory burst is pivotal for microbial killing, as superoxide and derived ROS (H_2_O_2_, HOCl, etc.) damage pathogens. Notably, the persistence of NOX2 activity is partially sustained by PI3K-dependent phosphoinositides that keep the complex assembled, but as those signaling lipids are metabolized, the oxidase activity will naturally attenuate [46].

Phagocytes also employ multiple negative regulatory mechanisms to fine-tune NOX2 activity and prevent excessive oxidative damage. One such mechanism is mediated by NRROS (“Negative Regulator of ROS”), an ER-resident protein that binds nascent gp91phox and promotes its degradation before it can assemble into active oxidase complexes [47]. Loss of NRROS leads to heightened ROS output in macrophages and neutrophils, enhancing pathogen clearance but at the cost of increased inflammatory tissue injury (for example, NRROS-deficient mice exhibit exacerbated neuroinflammation in an EAE model) [47]. At the gene regulation level, a recent functional genomics study identified transcriptional and metabolic “brakes” on the oxidative burst. The transcription factor RBP-J (Notch pathway mediator) was found to repress basal expression of multiple NOX2 subunit genes (Cybb encoding gp91phox, Ncf1 encoding p47phox, etc.), thereby limiting the amount of oxidase available [48]. In parallel, the glycolytic enzyme PFKL and the E3 ubiquitin ligase RNF145 were shown to curtail NADPH oxidase activity by restricting NADPH supply (via the pentose phosphate pathway) and by targeting oxidase subunits for degradation, respectively [49]. These layers of regulation ensure that the oxidative burst is robust yet self-limiting. On a faster timescale, post-activation feedback mechanisms can directly inhibit NOX2. For instance, cAMP-elevating signals (e.g., via β2-adrenergic receptors) activate PKA, which can phosphorylate the cytosolic C-terminus of gp91phox, inducing a conformational change that hinders further assembly of cytosolic subunits and reduces NADPH oxidase activity [50]. Likewise, proteolytic regulation has been observed: Matrix Metalloproteinase-2 (MMP-2) can cleave the extracellular loop of gp91phox, releasing a soluble peptide (termed sNOX2-dp) and in doing so, functionally incapacitating the oxidase [51]. Through such negative regulators, phagocytes prevent unchecked ROS production that could damage host tissues once microbes are cleared.

### 5.2. ROS Impact on NF-κB and MAPK Signaling

Beyond their direct microbicidal effects, ROS generated by NOX2 act as critical secondary messengers in immune signaling. Upon pathogen encounter, phagocyte receptors (Toll-like receptors, Fc receptors, integrins, etc.) trigger not only oxidase assembly but also activate inflammatory signaling pathways like NF-κB and MAPKs. Emerging evidence indicates these processes are interconnected: ROS can modulate the activity of various kinases and transcription factors, thereby influencing gene expression programs. In fact, acute increases in ROS levels have been shown to augment the activation of NF-κB and MAPK pathways in macrophages, leading to amplified production of pro-inflammatory cytokines [52,53]. Mechanistically, one way ROS enhances such signaling is by oxidizing redox-sensitive cysteine residues in signaling proteins—often inactivating protein tyrosine phosphatases, which tilts the balance toward kinase activation. For example, ROS can oxidize the thiol group of thioredoxin, causing its release from the apoptosis signal-regulating kinase 1 (ASK1). Freed from thioredoxin-mediated inhibition, ASK1 becomes active and phosphorylates downstream MAP2Ks, culminating in the activation of p38 MAPK and JNK cascades [54,55]. Through this pathway, a respiratory burst can swiftly activate p38 MAPK, which in turn phosphorylates targets like transcription factors (e.g., ATF-2, Elk-1) and downstream enzymes, ultimately driving the expression of inflammatory genes and cytokines. Similarly, NF-κB signaling can be sensitized by ROS: moderate levels of H_2_O_2_ have been reported to promote the activation of IκB Kinase (IKK) complex, accelerating the degradation of IκBα and the nuclear translocation of NF-κB, thereby upregulating genes for TNF-α, IL-1β, and other mediators [52,56]. Consistently, macrophage studies show that elevated ROS can increase NF-κB DNA-binding activity and enhance the transcription of inflammatory cytokines, linking the oxidative burst to heightened immune response [52].

It is important to note, however, that the influence of ROS on signaling is biphasic and highly concentration-dependent. While a transient or moderate ROS burst can act as a stimulatory signal, excessive or sustained ROS (and reactive nitrogen species, RNS) can become detrimental, triggering stress pathways or inducing immunosuppressive effects. High levels of ROS/RNS may directly damage signaling proteins or enforce post-translational modifications that blunt pro-inflammatory pathways. For instance, in conditions of chronic oxidative or nitrosative stress, NF-κB subunits can undergo S-nitrosylation on critical cysteine residues, which inhibits their DNA-binding capability and thus blocks NF-κB–dependent transcription [57,58]. Similarly, hyperoxidation of MAPK phosphatases can inactivate these regulatory enzymes, sometimes leading to uncontrolled kinase activity followed by a cell’s apoptotic shutdown. In T lymphocytes, which are less hardy against oxidative stress than phagocytes, an overabundance of ROS can paradoxically inhibit activation: experimental studies have shown that memory T cells exposed to high ROS fail to activate NF-κB and undergo apoptosis or anergy [59]. Therefore, cells maintain a delicate redox balance, often via antioxidant systems, to permit ROS-mediated signaling without incurring oxidative damage. This balance is exemplified by the need to have an initial ROS spike for signaling, followed by prompt engagement of antioxidant defenses to terminate the signal and prevent collateral harm.

### 5.3. Nrf2: Antioxidant Response and Feedback Regulation

Given the potential for self-damage by ROS, immune cells rely on redox-sensitive transcription factors to sense and adapt to changes in intracellular ROS levels. Central among these is NF-E2–related factor 2 (Nrf2), often called the master regulator of antioxidant responses. Nrf2 is normally sequestered in the cytoplasm by its inhibitor Keap1, which targets Nrf2 for proteasomal degradation. During a robust oxidative burst, ROS modify critical cysteine residues on Keap1, allowing Nrf2 to escape degradation and translocate into the nucleus. There, Nrf2 binds to antioxidant response elements (ARE) in the promoters of numerous detoxification and antioxidant genes. Through this mechanism, Nrf2 induces the expression of heme oxygenase-1 (HO-1), NAD(P)H quinone dehydrogenase 1 (NQO1), glutamate-cysteine ligase (GCL), glutathione peroxidase, superoxide dismutase, and other cytoprotective proteins [60]. The result is an elevated capacity to scavenge ROS and repair oxidative damage, which acts as a negative feedback loop to the initial NOX2-derived burst. In phagocytes, Nrf2 activation is crucial for limiting tissue damage, it ensures that while pathogens are being killed by ROS, the host cell’s own proteins and DNA are shielded as much as possible from oxidative harm. Consistently, Nrf2-deficient mice exhibit hyperinflammatory phenotypes; their macrophages produce excessive inflammatory cytokines and suffer elevated tissue injury due to insufficient antioxidant countermeasures [61].

Besides neutralizing ROS, Nrf2 has profound immunomodulatory effects. Nrf2-driven gene products not only restore redox homeostasis but also reshape signaling pathways. One notable outcome is the impact on macrophage polarization and inflammatory gene expression. Nrf2 activation skews macrophages toward a less inflammatory phenotype (akin to M2), in part by suppressing NF-κB-driven proinflammatory genes [61]. For example, Nrf2 can upregulate HO-1; the enzymatic products of HO-1 (carbon monoxide and bilirubin) are themselves signaling molecules that dampen NF-κB and inflammasome activity, thus reducing IL-1β and TNF-α secretion [60]. At a molecular level, crosstalk between Nrf2 and NF-κB serves as a pivotal node of redox–inflammation balance. Nrf2 and NF-κB can mutually antagonize each other’s activity through competition for transcriptional coactivators and other mechanisms [62]. For instance, active NF-κB has been shown to recruit histone deacetylase 3 (HDAC3) to Nrf2-regulated gene promoters, leading to deacetylation of Nrf2 and attenuation of its transcriptional activity [63]. Conversely, abundant Nrf2 in the nucleus can sequester limiting amounts of CREB-binding protein (CBP) away from NF-κB, thereby reducing NF-κB-mediated transcription [64]. Through such bidirectional interactions, a homeostatic equilibrium is maintained: during acute infection, NF-κB and other inflammatory pathways dominate to mount a defense, while Nrf2 is transiently restrained; as ROS accumulate, Nrf2 is unleashed to restore redox balance and put the brakes on inflammation. This interplay is critical for preventing chronic inflammation and oxidative tissue damage once a pathogen is cleared. The Nrf2 pathway is also a therapeutic target of interest—pharmacological Nrf2 activators (e.g., dimethyl fumarate) have been shown to reduce inflammatory damage in disorders by both enhancing antioxidant defenses and inhibiting NF-κB activity [61].

### 5.4. Redox Regulation in Lymphocytes and Dendritic Cells

While professional phagocytes (neutrophils, macrophages, monocytes) are the primary executors of the oxidative burst, other immune cells also exhibit redox-regulated signaling processes that echo those in phagocytes. T lymphocytes, for example, generate ROS as second messengers during activation. Upon T cell receptor (TCR) engagement by antigen-presenting cells, there is a rapid increase in ROS production from both mitochondria and a NOX2-like enzyme in T cells [52]. This burst of ROS is integral to T cell activation: it participates in the activation of transcription factors NF-κB, NFAT, and AP-1, and supports the IL-2 production and clonal expansion that follow [65]. The ROS transiently inhibit certain phosphatases (such as SHP-1 and PTEN) that normally dampen TCR signaling, thereby amplifying kinase signaling cascades needed for full T cell activation. If ROS generation is blocked (for instance, by strong antioxidants), TCR signaling is blunted and T cells do not proliferate robustly [52]. However, as noted earlier, an overabundance of ROS is harmful to lymphocytes. T cells have lower antioxidant reserves compared to phagocytes; thus, excessive ROS can lead to T cell dysfunction or death. In line with this, sustained high ROS in the T cell environment encourages apoptotic pathways and can drive T cells into an unresponsive state (exhaustion). Indeed, studies have observed that chronically elevated ROS levels (or deficient antioxidant enzymes) in T cells correlate with upregulation of inhibitory receptors like PD-1 and a failure to maintain NF-κB and metabolic signaling, culminating in impaired effector function [52]. On the other hand, regulatory T cells (Tregs) appear more resistant to oxidative stress, in part because Nrf2 is induced during TCR stimulation of naive T cells and biases their differentiation toward the Treg lineage at the expense of Th1/Th17 lineages [66]. Thus, redox signals help shape T cell fate decisions, a moderately oxidative milieu favors certain immune responses, whereas an overly oxidative environment can suppress T cell immunity and promote regulatory or suppressive phenotypes.

Dendritic cells (DCs), which are phagocytic antigen-presenting cells, also leverage NOX2-derived ROS for functions beyond pathogen killing. DCs assemble the NADPH oxidase complex on phagosomal membranes, and the ROS produced help to control phagosomal pH and proteolysis rate, which is pivotal for antigen processing. ROS consumption of protons raises the phagosomal pH, preventing overly rapid acidification [67]. This is beneficial for cross-presentation of antigens: a higher pH in early phagosomes preserves antigenic proteins longer, allowing DCs to process and load peptides onto MHC class I for CD8^+^ T cell priming [68]. Indeed, human DCs with defective NOX2 activity have overly acidic phagosomes and show impaired cross-presentation of exogenous antigens [69]. Moreover, ROS in DCs can act as maturation signals. During infection, TLR stimulation in DCs triggers a modest oxidative burst that feeds into signaling pathways (such as p38 MAPK and NF-κB), promoting the production of cytokines like IL-12 and the upregulation of costimulatory molecules [70]. If DCs are treated with potent antioxidants, their maturation and cytokine secretion are dampened [71], leading to suboptimal T cell activation. Therefore, a degree of ROS is necessary for DCs to acquire a fully stimulatory phenotype.

In summary, NADPH oxidase-derived ROS orchestrate a wide array of immune cell functions: they directly destroy pathogens, but also serve as signaling molecules that regulate immune cell activation, gene expression, and intercellular communication. In phagocytes, the NOX2 complex is tightly regulated by phosphorylation events, small GTPases, and feedback inhibitors to ensure ROS are produced only when needed and in appropriate amounts. These ROS then modulate key signaling pathways, activating NF-κB and MAPKs to amplify microbicidal and inflammatory responses, while eventually triggering Nrf2 and other counter-regulatory mechanisms to prevent excessive inflammation. Redox-sensitive signaling extends to other immune cells as well, influencing T cell and dendritic cell responses in the broader immune network. A deeper understanding of this redox regulation not only illuminates fundamental immune biology but also reveals potential therapeutic targets (for instance, boosting host defense by modulating NOX2 activity or tempering inflammation by enhancing Nrf2 signaling) [72]. Such insights underscore the dual nature of ROS in immunity, functioning as indispensable defenders and as careful modulators of immune signaling.

## 6. Clinical Relevance and Pathophysiological Dysregulation

An adequate level of ROS generated by the phagocyte NADPH oxidase complex (NOX2) is essential for anti-infective immunity. Both deficiency and uncontrolled excess of ROS have serious clinical consequences. Inherited NOX2 deficiency produces a severe immunodeficiency, chronic granulomatous disease (CGD), characterized by recurrent bacterial and fungal infections, whereas uncontrolled ROS release causes inflammatory tissue injury and organ dysfunction [35]. Below, we discuss the clinical implications of NOX2 dysregulation in human diseases, highlighting both the effects of ROS deficiency and those of ROS excess, with immunological and translational relevance.

### 6.1. NOX2 Deficiency: Chronic Granulomatous Disease and Susceptibility to Infections

Chronic granulomatous disease (CGD) illustrates the impact of ROS deficiency on human health. CGD is a rare primary immunodeficiency caused by mutations in genes of the NADPH oxidase complex, for example, the CYBB gene that encodes the catalytic subunit NOX2/gp91phox [73]. These mutations block the phagocyte respiratory burst, making them unable to produce superoxide and H_2_O_2_ for microbial killing [74]. Clinically, most patients with CGD develop, from childhood, severe recurrent infections with pathogenic bacteria and fungi, often life threatening [75]. Typical infections include recurrent pneumonias, lung abscesses, lymphadenitis, and septicemias caused by organisms such as *Staphylococcus aureus*, *Salmonella* spp., *Aspergillus*, and *Candida* [76]. The lack of ROS in phagocytes compromises the efficient destruction of pathogens, allowing their survival and dissemination in the body [77].

Beyond intractable infections, CGD is also characterized by hyperinflammatory tendencies and granuloma formation. The immune system attempts to isolate uncleared microbes through the aggregation of macrophages and lymphocytes, resulting in inflammatory granulomas in various organs, for example, gastrointestinal granulomatosis that can obstruct the esophagus or intestine [35]. Many patients with CGD develop sterile tissue lesions and chronic inflammation, such as granulomatous colitis resembling Crohn disease, suggesting that ROS also have an immunomodulatory role in limiting exaggerated inflammatory reactions [78]. Paradoxically, although ROS are lacking, inflammation is not absent, rather, ROS deficiency can dysregulate the immune system’s negative feedback pathways, leading to autoinflammatory phenomena and uncontrolled activation of innate immunity [79]. This autoinflammation associated with NOX2 deficiency underscores the importance of ROS in maintaining immune tolerance and preventing exaggerated responses. Fortunately, early recognition of CGD has enabled the implementation of prophylactic measures, antibiotics or antifungals, interferon γ, and curative treatment through hematopoietic stem cell transplantation or gene therapy, highlighting the translational potential of understanding this disease.

### 6.2. Uncontrolled Excess of ROS in Sepsis

In sepsis, which represents a systemic inflammatory response to generalized infections [80,81], the imbalance between ROS production and the capacity of host antioxidant systems promotes systemic oxidative stress, amplifying inflammation and impairing cellular function. Excess ROS disrupt the microcirculation, promote coagulopathy, and contribute to organ dysfunction.

In this setting, excessive activation of neutrophils and monocytes by microbial toxins and proinflammatory cytokines induces an exaggerated oxidative burst; massive amounts of ROS are released both intracellularly, within phagolysosomes, and extracellularly, through intense activation of phagocyte NADPH oxidases and other sources, mitochondria, xanthine oxidase, etc. [82]. This excess of free radicals exceeds the capacity of antioxidant systems, glutathione, catalase, superoxide dismutase, and leads to a sustained oxidative imbalance [82]. A vicious cycle of inflammation is thus created: excess ROS consume endogenous antioxidants and worsen the release of proinflammatory cytokines, recruiting more immune cells and generating even more ROS [82].

The pathophysiological consequences of the ROS torrent in sepsis are profound. ROS attack membrane lipids, proteins, and nucleic acids of host cells, causing diffuse tissue injury in the lungs, liver, kidneys, and other vital organs, including the vascular endothelium.

In the endothelium, oxidative stress disrupts the protective glycocalyx and activates endothelial cells, which express adhesion molecules, ICAM-1 and selectins, and procoagulant factors [83]. The result is leukocyte and platelet adhesion to the vessel wall and the release of tissue factor, triggering disseminated intravascular coagulation, widespread microthrombus formation and consumption of coagulation factors [84]. Neutrophils stimulated by ROS also release proteolytic enzymes and form extracellular DNA traps, NETs, which can aggravate endothelial injury and induce microvascular thrombi. Together, these events lead to tissue hypoperfusion, multiorgan dysfunction, and septic shock. In later stages, sepsis can deplete immune reserves, including the oxidative capacity of phagocytes, leaving the patient vulnerable to opportunistic infections, a dangerous alternation between initial hyperinflammation and secondary immunosuppression [85].

In endothelial cells, NOX2-derived ROS not only exert direct oxidative effects but also initiate a self-amplifying redox loop through mitochondria. Experimental models of sepsis have shown that NOX2 activation increases mitochondrial electron leakage, generating additional ROS that further stimulate NOX2, a bidirectional process termed ROS-induced ROS release [86]. This crosstalk between NOX2 and mitochondrial sources enhances endothelial dysfunction, promotes barrier leakage, and augments microvascular thrombosis [87]. Although other vascular NOX isoforms may contribute, current evidence indicates that NOX2-dependent mitochondrial amplification is a key driver of redox imbalance in septic endothelial injury.

**Figure 2:**
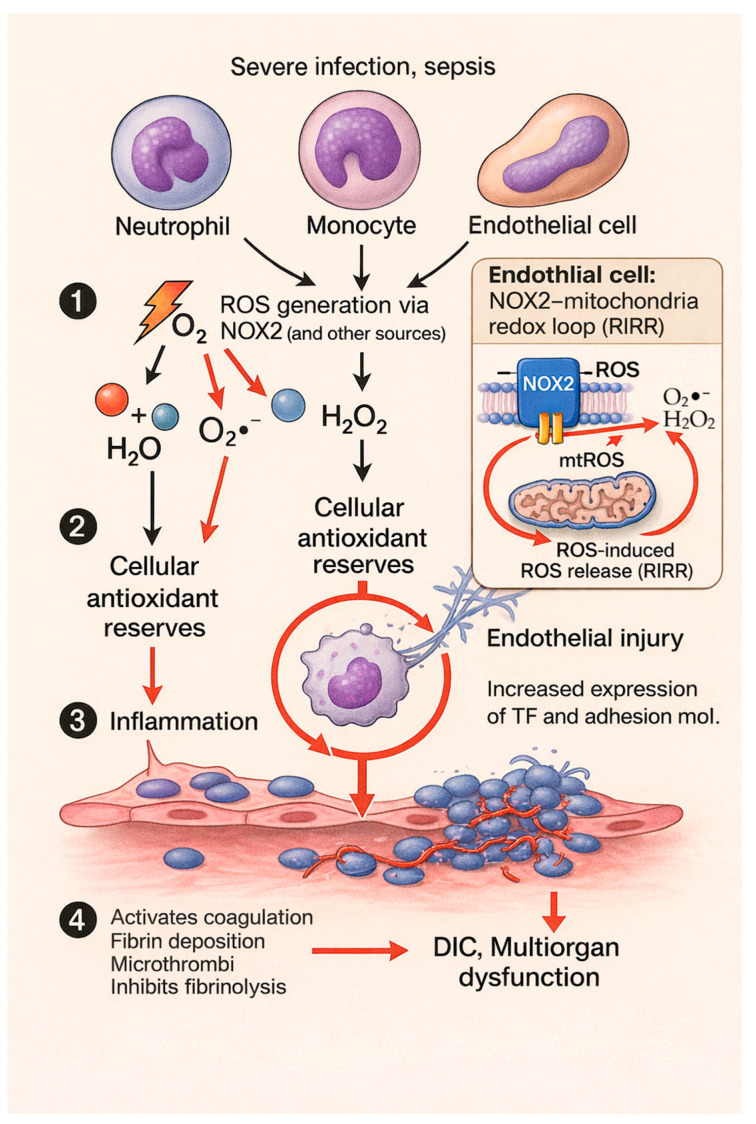
Severe infections, sepsis, induce the activation of neutrophils, monocytes, and endothelial cells, resulting in excessive ROS production via NADPH oxidase (NOX2) and other sources. (1) Overproduction of superoxide and H_2_O_2_ overwhelms cellular antioxidant systems, leading to oxidative stress and (2) depletion of intracellular antioxidant reserves. (3) Sustained ROS drive inflammation and endothelial injury. In endothelial cells, NOX2-derived ROS engage a self-amplifying redox loop involving mitochondrial ROS production (ROS-induced ROS release, RIRR). (4) Endothelial dysfunction promotes tissue factor expression, coagulation activation, microthrombus formation, and impaired fibrinolysis, contributing to disseminated intravascular coagulation and multiorgan dysfunction.

The broader pathophysiological framework of NOX2-derived ROS–driven endothelial injury and coagulation imbalance in sepsis is illustrated in Figure 2.

Understanding the role of ROS in sepsis has important therapeutic implications. On the one hand, ROS remain necessary for microbial killing, on the other hand, attenuating oxidative injury has become a target in the management of severe sepsis. Clinical studies are investigating the administration of systemic antioxidants, for example, high dose vitamin C and *N*-acetylcysteine, to counteract oxidative stress in sepsis [82]. In addition, inhibitory molecules of NADPH oxidase, such as apocynin or more selective inhibitors, could limit ROS production in the hyperinflammatory phase. Such interventions, however, must be carefully dosed so as not to completely compromise antimicrobial defense. In principle, targeting the NOX2, ROS pathway represents a promising strategy to reduce excessive inflammation and tissue damage in sepsis [88], complementing standard anti infectious therapies, antibiotics and hemodynamic support. Fine balancing of the oxidative response, neither too weak nor excessive, is therefore essential to improve outcomes in patients with sepsis.

### 6.3. Redox Dysregulation in Chronic Inflammation and Autoimmunity

Chronic oxidative stress contributes to the pathogenesis of many chronic inflammatory and autoimmune diseases. Persistent, excessive generation of ROS produces a state of low-grade inflammation in tissues, through continuous activation of proinflammatory signaling pathways, for example, NF-κB and the NLRP3 inflammasome, and through direct injury to parenchymal cells. Over time, this process leads to cumulative tissue damage and fibrosis, being implicated in diseases such as atherosclerosis, diabetic nephropathy, rheumatoid arthritis, neurodegeneration, and even cancers associated with inflammation [20]. For example, high levels of ROS have been correlated with endothelial dysfunction and vascular inflammation that promote diabetes and atherogenesis, similarly, chronic oxidative stress can induce mutations and proliferative dysregulation related to cancer [20]. In addition, large amounts of ROS can modify self-structures, oxidation of proteins and DNA, generating neoepitopes and the breakdown of immunological tolerance, which is a mechanism proposed in the etiology of some autoimmune diseases [20].

On the other hand, redox dysregulation can also cause insufficient oxidative signaling, with paradoxical effects on inflammation. Recent evidence suggests that ROS produced by NOX2 play a subtle role in regulating the adaptive immune response and maintaining tolerance to self [88]. In the absence of an adequate level of ROS, immune cells may miss the signals to stop inflammation or to undergo apoptosis of activated cells, resulting in an environment conducive to autoimmunity and uncontrolled inflammation. This situation is seen in extreme form in patients with CGD, in addition to infections, they present autoinflammatory phenomena, such as chronic granulomatosis, precisely because of the lack of ROS that would normally contribute to closing the inflammatory response [88]. Even partial forms of oxidative deficiency, hypomorphic mutations of NOX2 subunits, have been associated with susceptibility to autoimmune diseases, for example, systemic lupus erythematosus, in some studies, underscoring the role of ROS in maintaining immune homeostasis. Thus, an optimal redox balance is critical not only in acute infections, but also in the long term, too little ROS can favor chronic inflammation and immune self-aggression, whereas too much ROS causes tissue injury and promotes a vicious inflammatory cycle.

### 6.4. Redox Dysfunction in Viral Infections and COVID-19

Viral infections also trigger significant disturbances of the oxidative balance. Many viruses induce ROS production in host cells either directly, through activation of oxidases and mitochondria, or indirectly, through induction of pro-oxidant cytokines and inhibition of antioxidant enzymes [89]. The consequence is marked oxidative stress that can affect the antiviral immune response and can cause damage to infected tissues. For example, increased ROS in infection with influenza virus or flaviviruses, dengue and Zika, has been correlated with destruction of infected cells through apoptosis, dysfunction of endothelial barriers, and amplification of local inflammation, contributing to the severity of clinical manifestations [89]. In addition, an excessive level of ROS can suppress certain antiviral signaling pathways, for example, chronic oxidative stress can inhibit the efficient activation of transcription factors for type I interferon, weakening the control of viral replication. Thus, viruses often exploit host redox, an insufficient oxidative response can permit uncontrolled viral replication, whereas an excessive response causes inflammation and collateral damage [90].

A particular case, infection with SARS-CoV-2 (COVID-19) has strongly highlighted the role of redox dysregulation in the pathogenesis of a viral disease. Severe forms of COVID-19 are characterized by a cytokine storm and massive activation of immune and endothelial cells. This hyperinflammatory milieu leads to activation of the NOX2 NADPH oxidase in endothelium and in alveolar macrophages, with excessive production of superoxide and H_2_O_2_ at the pulmonary level [91]. Studies show that NOX2 is overstimulated by inflammatory cytokines, IL-6, TNF-α, in the lungs of patients with severe COVID-19, generating ROS that compromise endothelial function and increase capillary permeability [91]. ROS drive activation of the NLRP3 inflammasome and the expression of endothelial adhesion molecules, creating a proinflammatory phenotype that favors excessive neutrophil recruitment and the formation of pulmonary microthrombi [89]. Indeed, a state of severe oxidative stress has been proposed as a hallmark of critical COVID-19, contributing to acute respiratory distress syndrome (ARDS) and to the thromboembolic complications observed in these patients.

Given this involvement, targeting the NOX2 and ROS axis in COVID-19 is being explored as an adjunct therapeutic approach. One promising example is the inhibitory peptide PIP-2, designed to prevent NOX2 activation by inhibiting the phospholipase iPLA2 required for NOX2 subunit translocation [92]. In preclinical models, pretreating endothelial cells with PIP-2 led to a significant reduction in ROS production induced by serum from patients with severe COVID-19, thus dampening the proinflammatory response, endothelial activation markers (ICAM-1, P-selectin), cytokine secretion, and cell death were markedly decreased compared with control [93]. These findings suggest that selective inhibition of NADPH oxidase could protect vessels and tissues from the oxidative “storm” in COVID-19, improving disease course. Of course, clinical application requires caution, antiviral immunity depends in part on ROS for the destruction of infected cells. Nevertheless, in cases of critical COVID-19, where the host response becomes disproportionately harmful, redox modulation, through systemic antioxidants, NOX2 inhibitors, or enhancement of the NRF2 antioxidant pathway, represents a major area of interest [94].

In conclusion, NOX2 dysfunction and dysregulated ROS production underpin a range of pathologies, from recurrent infections to chronic inflammation and acute hyperinflammatory syndromes. ROS deficiency, as in CGD, compromises antimicrobial defense and can trigger autoinflammation, highlighting the crucial role of ROS in immune regulation [35]. At the opposite end, excess ROS, as seen in sepsis or COVID-19, causes severe tissue injury and immune dysfunction, underscoring the need to temper the oxidative response in such conditions [20,82]. A better understanding of these mechanisms has opened valuable translational avenues, from therapies that augment oxidase function, hematopoietic stem cell transplantation and gene therapy in CGD, to antioxidant therapies and NOX inhibitors for diseases driven by oxidative stress. Maintaining the redox balance of the immune response is, in essence, a key target for future therapeutic interventions, with the potential to improve both infection control and the reduction in collateral inflammatory damage. Citing the toxicological adage “dosis facit venenum,” the dose makes the poison, and in the context of ROS, both too little and too much can be detrimental to health [20]. Therefore, the fine regulation of NOX2 NADPH oxidase and ROS production remains a major area of interest, with direct clinical implications for human infectious, autoimmune, and inflammatory diseases.

## 7. Therapeutic Modulation of NADPH Oxidase and ROS

Excessive NADPH oxidase (NOX) activity and uncontrolled ROS can drive tissue injury, yet ROS are indispensable for pathogen killing. Thus, therapies must fine-tune ROS levels, not simply abolish them [95,96]. Recent research has explored multiple experimental strategies, from selective NOX2 inhibitors to Nrf2 pathway activators and redox-modulating peptides, aiming to modulate NOX-derived ROS while preserving antimicrobial defense. A summary of experimental strategies targeting NOX2 is provided in Supplementary Table S1.

### 7.1. Selective NOX2 Inhibitors

One direct approach is to inhibit NOX2, the phagocyte NADPH oxidase. Apocynin is a classic NOX2 inhibitor derived from plants. It blocks assembly of the multi-subunit NOX2 complex by preventing cytosolic subunits (p47phox, p67phox) from translocating to the membrane [97]. Notably, apocynin acts as a prodrug: in neutrophils, myeloperoxidase converts apocynin monomers into active dimeric forms (diapocynin) that inhibit NOX2 and lower ROS production [97]. In numerous animal models of inflammation, apocynin treatment curbed oxidative damage and improved outcomes, for example, reducing vascular inflammation, neuroinflammation, and even improving lung function in COPD patients [98]. However, apocynin’s efficacy can vary by cell type (it is a potent NOX2 inhibitor in phagocytes, but in non-phagocytic cells it may act more as an antioxidant scavenger or even pro-oxidant) [97]. Its need for metabolic activation and modest potency have driven the search for more selective drug-like inhibitors.

A breakthrough in selective NOX2 inhibition was achieved with GSK2795039, a small molecule identified by GlaxoSmithKline, which acts as a competitive inhibitor of NOX2 by competing for NADPH binding, displaying high selectivity over other NOX isoforms and related enzymes, and potently suppressing superoxide production and oxygen consumption by NOX2 in cell-based assays [99]. Importantly, GSK2795039 was the first small molecule to demonstrate in vivo NOX2 inhibition, as it abolished ROS production in a mouse paw inflammation model and attenuated disease severity in acute pancreatitis [99]. These results validate that targeting NOX2 enzymatic activity can protect tissues from inflammatory damage. Derivatives of GSK2795039 are now being optimized: for instance, modifying that scaffold led to NCATS-SM7270, an even more specific NOX2 inhibitor that showed neuroprotective effects in traumatic brain injury models [100]. Beyond NOX2, inhibitors targeting other NOX isoforms (e.g., the dual NOX1/4 inhibitor setanaxib) have entered clinical trials for fibrotic diseases, underscoring the therapeutic interest in NADPH oxidase modulation. It should be noted that a pan-NOX flavoprotein inhibitor, diphenyleneiodonium (DPI), has long been used in research but is too non-specific and toxic for therapy. In contrast, newer inhibitors like GSK2795039 aim to spare host defense by selectively dampening pathological NOX2 activation rather than abolishing it entirely.

### 7.2. Nrf2 Pathway Activators and Antioxidant Therapies

An indirect strategy to modulate ROS is boosting the cell’s antioxidant defenses via the Nrf2 pathway. Nrf2 (nuclear factor erythroid 2-related factor 2) is a master transcription factor that upregulates numerous antioxidant and cytoprotective genes (e.g., heme oxygenase-1, glutathione-synthesizing enzymes, NAD(P)H: quinone oxidoreductase-1). Under stress, Nrf2 dissociates from its inhibitor Keap1 and translocates to the nucleus to activate an array of antioxidant response element (ARE)-driven genes [101]. By enhancing ROS scavenging and detoxification, Nrf2 activators can counterbalance excessive NOX-derived oxidants without directly inhibiting NOX enzymes.

Dimethyl fumarate (DMF) is a prime example. Originally a psoriasis drug, DMF is now approved for multiple sclerosis, where it exerts anti-inflammatory and neuroprotective effects partly through Nrf2 activation [102]. DMF is an electrophilic molecule that alkylates Keap1 cysteines, stabilizing Nrf2. This leads to raised intracellular glutathione, HO-1, and other antioxidants, broadly dampening oxidative injury. Interestingly, DMF’s benefits may extend beyond Nrf2, it also modulates immune signaling and metabolism, but its ability to induce the Nrf2/ARE pathway is central to its cytoprotective action [102]. Preclinically, DMF and its metabolite monomethyl fumarate have shown efficacy in models of neurodegeneration, ischemia, and infection, by reducing pro-inflammatory cytokines and oxidative damage.

Sulforaphane, a natural isothiocyanate from broccoli, is another well-studied Nrf2 activator. Sulforaphane reacts with Keap1 to free Nrf2, triggering robust antioxidant enzyme induction. In macrophages infected with *Mycobacterium tuberculosis*, sulforaphane upregulated Nrf2 target genes (HO-1, NQO1) and significantly mitigated oxidative stress, lowering intracellular ROS levels [101,103,104]. This Nrf2-driven antioxidant boost also curbed NLRP3 inflammasome activation and pyroptotic cell death in the infected macrophages [101]. In other models, sulforaphane has been shown to attenuate endotoxin-induced inflammation and reduce neutrophil ROS release [105]. These findings suggest that enhancing endogenous antioxidant responses can protect tissues during infection or inflammation, effectively “mopping up” excess ROS, while still allowing phagocytes to produce some ROS for microbial killing. Besides DMF and sulforaphane, other Nrf2 activators (e.g., bardoxolone methyl, oleanolic acid derivatives, and itaconate analogs) are under investigation for conditions ranging from sepsis to organ fibrosis [102]. The appeal of Nrf2-based therapy is its ability to restore redox balance without directly suppressing the microbicidal ROS burst. The challenge, however, is that chronic over-activation of Nrf2 might interfere with immune signaling or promote cancer cell survival, so timing and dosing require careful control.

### 7.3. Redox-Modulating Peptides

An emerging therapeutic avenue involves peptides that modulate redox enzymes or NOX2 assembly. One such innovative agent is the PLA_2_-inhibitory peptide 2 (PIP-2), a nine amino acid peptide derived from lung surfactant protein A. PIP-2 targets peroxiredoxin 6 (Prdx6), a bifunctional enzyme that provides a crucial “trigger” for NOX2 activation. Prdx6 has a calcium-independent phospholipase A_2_ (aiPLA_2_) activity that generates lysophospholipids, which in turn activate the small GTPase Rac, an essential co-factor for NOX2 assembly [106]. By binding Prdx6 and selectively inhibiting its PLA_2_ activity (while sparing its peroxidase function), PIP-2 effectively blocks the upstream signal needed for NOX2 complex formation [106]. The result is a potent indirect inhibition of NOX2-driven ROS production. In isolated mouse lungs, PIP-2 almost completely ablated angiotensin II-induced superoxide generation (mimicking the effect of NOX2 knockout) [104]. Impressively, in a mouse acute lung injury model induced by intratracheal LPS (endotoxin), PIP-2 demonstrated dramatic protective effects: a single dose given before LPS prevented the spike in lung ROS and halted neutrophil infiltration, protein leakage, and lipid peroxidation that characterize acute lung injury [107]. Even when administered 12–16 h after LPS exposure (to simulate a therapeutic scenario), PIP-2 reversed ongoing lung injury, restoring lung histology and reducing inflammatory markers to near-normal levels [107]. In a more sepsis model (lethal endotoxemia), repeated dosing of PIP-2 led to an 83% survival rate vs. 0% in controls, indicating profound rescue from inflammatory lung damage and multi-organ failure [108]. These outcomes underscore the promise of redox-modulating peptides like PIP-2 in taming hyperinflammation. Notably, PIP-2’s mechanism leaves basal phagocyte functions intact, it specifically prevents pathologic NOX2 over-activation by LPS/AngII without poisoning the NOX2 enzyme itself [108]. This specificity could translate to fewer immunosuppressive side effects compared to broad antioxidants.

Beyond PIP-2, other peptide-based NOX2 inhibitors have been explored. A cell-permeable peptide mimicking the NOX2 organizing subunit (sometimes called gp91ds-TAT) was shown in earlier studies to disrupt p47phox binding to NOX2, thereby reducing superoxide bursts. While gp91ds-TAT is more a research tool than a therapeutic candidate, it proved that targeting protein–protein interactions within the NOX2 complex is feasible. Similarly, the PIP peptide series (PIP-1, PIP-2, PIP-3) illustrates that short peptides can selectively modulate redox signaling upstream. Delivering peptides can be challenging (PIP-2 was formulated in liposomes for in vivo use), but advancements in peptide stability and delivery (nano-carriers, peptide mimetics) could overcome these hurdles.

### 7.4. Balancing ROS: Dosing Challenges and Future Directions

Therapeutic ROS modulation is a double-edged sword. Insufficient ROS generation can leave the host immunocompromised, whereas excessive ROS causes collateral damage. The extremes are illustrated by disease states: on one hand, patients with chronic granulomatous disease (inherited NOX2 deficiency) suffer recurrent infections and granulomas due to an inability to produce ROS [4]. On the other hand, sustained NOX2 hyperactivity contributes to chronic inflammatory pathology (atherosclerosis, neurodegeneration, etc.) [4]. Therefore, any therapy targeting NOX2/ROS must strike a careful balance between preserving antimicrobial defense and preventing tissue injury. Achieving this balance involves dose and timing optimization. For instance, completely shutting off NOX2 throughout an infection could be dangerous, but temporarily dampening an excessive respiratory burst in sepsis or ARDS might save lives. Strategies like PIP-2’s delayed post-insult administration exemplify how timing can mitigate damage without undermining early pathogen clearance. Likewise, partial inhibition or organ-targeted delivery (e.g., inhaled NOX inhibitors for pulmonary inflammation) could limit systemic effects.

Another consideration is that ROS have nuanced roles in immune signaling beyond pathogen killing. Low-level ROS modulate cytokine production and inflammatory cell recruitment [94]. Thus, “dosing ROS” in therapy may require titrating treatments to avoid quenching these signaling functions entirely. It should also be noted that inhibition of NOX2 may shift redox signaling toward other ROS sources, such as mitochondrial ROS or NOX1/4 in non-phagocytic cells, although compensatory mechanisms are still incompletely understood and were beyond the primary scope of this NOX2 centered review. One promising concept is combination approaches: pairing a NOX2 inhibitor with an antimicrobial or immune stimulant, to ensure infections are controlled even as oxidative damage is reduced. Additionally, some existing drugs indirectly modulate NOX2, for example, statins (HMG-CoA reductase inhibitors) prevent prenylation of Rac GTPase, thereby impairing NOX2 assembly. Simvastatin has been shown to reduce NOX2-mediated ROS and improve outcomes in mouse endotoxemia lung injury [106]. This hints that clinicians might repurpose adjuvant therapies (like statins or angiotensin receptor blockers) to mildly restrain NOX2 in hyperinflammatory states, without complete immunosuppression.

In summary, the past decade has yielded innovative strategies to therapeutically modulate NOX-derived ROS. Selective NOX2 inhibitors (apocynin analogues, GSK2795039 and successors) directly target the enzyme’s ROS output [99]. Nrf2 pathway activators (DMF, sulforaphane) bolster endogenous antioxidants to buffer ROS surges [101]. Redox peptides like PIP-2 interrupt the molecular triggers of NOX2 activation, offering a precision tool to prevent runaway oxidative bursts [106]. Each approach carries distinct advantages and challenges, but all converge on a common goal: taming harmful ROS excess while preserving the antimicrobial ROS toolkit. Moving forward, refining these therapies, perhaps through smart drug delivery, patient-specific dosing, or real-time monitoring of oxidative markers, will be crucial. By threading the needle between host defense and immunopathology, NADPH oxidase modulators hold significant therapeutic potential in infections and inflammatory diseases of the future.

## 8. Challenges and Future Perspectives

Despite progress in understanding NADPH oxidase (NOX2) and the role of ROS in antimicrobial defense, numerous challenges remain that hinder the therapeutic translation of this knowledge. The main current limitations include the lack of clinically approved inhibitors. At present, there are no specific NADPH oxidase inhibitors authorized for human use, and only one compound, setanaxib, has reached the stage of clinical trials [109]. This absence reflects the difficulties in developing agents that directly target NOX2.

Another major issue is the difficulty of modulating ROS without compromising antimicrobial defense. NOX2-derived ROS have a dual function: in excess, they cause oxidative stress and tissue injury, while in total absence, as in chronic granulomatous disease, they lead to severe recurrent infections [110]. Thus, nonspecific antioxidant interventions may undermine immunity and have proven ineffective in clinical trials, highlighting the need for fine regulation of NOX2 activity.

There are also discrepancies between animal models and human pathology. Much of the current understanding of NOX2 and oxidative stress comes from preclinical models that do not fully reproduce the complexity of human disease. Many antioxidant therapies or NOX inhibitors that have shown benefits in animals have failed to improve patient outcomes [111], underscoring translational gaps between experimental studies and human clinical settings.

Another limitation is the lack of standardized redox biomarkers. There are no universally accepted biomarkers for quantifying oxidative stress or NOX2 activity in patients. Current measurements of oxidative damage, such as lipid peroxidation products or protein oxidation markers, provide only a cumulative picture without capturing the real-time dynamics of oxidative stress [112]. This variability and lack of standardization hinder the evaluation of the efficacy of redox therapeutic interventions.

Additionally, there are major challenges in real-time monitoring of oxidative stress. Technologies for continuous in vivo detection of ROS remain limited. Without sensitive methods to track ROS fluctuations in real time, interventions cannot be optimally adjusted, and ROS-dependent pathological mechanisms remain incompletely characterized.

In the future, overcoming these obstacles will require the development of selective and safe NOX2 inhibitors that reduce pathological ROS production while preserving antimicrobial function, the identification of reliable and standardized redox biomarkers for clinical monitoring [113], and improved preclinical models that accurately reflect human responses. Cutting-edge approaches such as ROS biosensors or redox imaging, along with combined therapeutic strategies, may offer promising directions for harnessing the therapeutic potential of NADPH oxidase with an improved safety and efficacy profile.

## 9. Conclusions

ROS derived from NADPH oxidase, particularly via NOX2, are a pivot of antimicrobial defense, providing direct pathogen killing and finely tuning inflammatory signaling pathways. The evidence integrated in this review shows that the effectiveness of the oxidative response depends on strict spatiotemporal regulation of the NOX2 complex and on crosstalk with the NF-κB, MAPK, and Nrf2 networks. Both ROS deficiency (e.g., CGD) and uncontrolled excess (e.g., sepsis, severe forms of COVID-19) lead to major clinical consequences, underscoring the principle that “the dose makes the poison” as applied to immune redox. From a therapeutic perspective, experimental strategies, including selective NOX2 inhibitors and peptides that block oxidase assembly, as well as Nrf2 activators, outline the possibility of dosing ROS to mitigate tissue injury without compromising antimicrobial defense. Important barriers remain, including the lack of approved inhibitors, standardized redox biomarkers, and real-time in vivo monitoring tools. Combined approaches and personalized redox medicine, supported by advanced sensors and imaging, will be essential to translate these concepts into clinical benefit.

## Data Availability

The data that support the findings of this study are available in this article, and further inquiries can be directed to the corresponding author.

## Supplementary Materials

The following supporting information can be downloaded at: https://www.mdpi.com/article/10.3390/antiox15010055/s1, Table S1: Experimental strategies targeting NOX2 and redox modulation in infection and inflammation. References [114,115,116,117,118] are cited in the Supplementary Materials.
