# Supplementary material for: Phagocyte NADPH Oxidase NOX2-Derived Reactive Oxygen Species in Antimicrobial Defense: Mechanisms, Regulation, and Therapeutic Potential—A Narrative Review
Source: Antioxidants (Basel). 2025 Dec 31;15(1):55. doi: 10.3390/antiox15010055 (PMC12837977; doi:10.3390/antiox15010055)
Supplement: Supplementary file 1 [file antioxidants-15-00055-s001.zip › antioxidants-4001989-supplementary.pdf]

Table S1. Experimental strategies targeting NOX2 activity and ROS signaling

Strategy	Example	Mechanism	Evidence	Key limitation	Reference
Phenolic inhibitor	Apocynin	Inhibits assembly of the NOX2 complex by preventing p47^phox translocation to the membrane	Preclinical models (in vivo inflammation and vascular models)	Low specificity, requires MPO-dependent activation	Heumüller et al., 2008, [1]
Peptide inhibitor	gp91ds-tat	Competitive peptide that blocks the interaction between gp91^phox (NOX2) and p47^phox, preventing oxidase assembly	In vivo and ex vivo models	Delivery, stability, not clinically approved	Rey et al., 2001, [2]
Small molecules	VAS2870	Pan-NOX inhibitor, interferes with NADPH oxidase activity including NOX2	Preclinical animal and ex vivo vascular models	Limited NOX isoform selectivity	Wind et al., 2010, [3]
Nrf2 activation	Sulforaphane	Enhances Nrf2-mediated antioxidant gene expression	Cellular and animal models	Does not directly target NOX2	Townsend et al., 2015, [4]
Genetic models	NOX2 knockout	Genetic ablation of NOX2 activity, proof-of-concept for pathogenic role of NOX2-derived ROS	Animal genetic models (proof-of-concept)	Not translatable to humans	Pollock et al., 1995, [5]

References

1. Heumüller, S.; Wind, S.; Barbosa-Sicard, E.; Schmidt, H.H.; Busse, R.; Schröder, K.; Brandes, R.P. Apocynin is not an inhibitor of vascular NADPH oxidases but an antioxidant. *Hypertension* **2008**, *51*, 211–217. https://doi.org/10.1161/HYPERTENSIONAHA.107.100214.

2. Rey, F.E.; Cifuentes, M.E.; Kiarash, A.; Quinn, M.T.; Pagano, P.J. Novel competitive inhibitor of NAD(P)H oxidase assembly attenuates vascular O_2^- and systolic blood pressure in mice. *Circ. Res.* **2001,** *89*, 408–414. https://doi.org/10.1161/hh1701.096037.

3. Wind, S.; Beuerlein, K.; Armitage, M.E.; Taye, A.; Kumar, A.H.; Janowitz, D.; Neff, C.; Shah, A.M.; Wingler, K.; Schmidt, H.H. Oxidative stress and endothelial dysfunction in aortas of aged spontaneously hypertensive rats by NOX1/2 is reversed by NADPH oxidase inhibition. *Hypertension* **2010**, *56*, 490–497. https://doi.org/10.1161/HYPERTENSIONAHA.109.149187.

4. Townsend, B.E.; Johnson, R.W. Sulforaphane induces Nrf2 target genes and attenuates inflammatory gene expression in microglia from brain of young adult and aged mice. *Exp. Gerontol.* **2016**, *73*, 42–48. https://doi.org/10.1016/j.exger.2015.11.004.

5. Pollock, J.D.; Williams, D.A.; Gifford, M.A.; Li, L.L.; Du, X.; Fisherman, J.; Orkin, S.H.; Doerschuk, C.M.; Dinauer, M.C. Mouse model of X-linked chronic granulomatous disease, an inherited defect in phagocyte superoxide production. *Nat. Genet.* **1995**, *9*, 202–209. https://doi.org/10.1038/ng0295-202.
